# High-Purity Preparation of HSV-2 Vaccine Candidate ACAM529 Is Immunogenic and Efficacious *In Vivo*

**DOI:** 10.1371/journal.pone.0057224

**Published:** 2013-02-26

**Authors:** Sophia T. Mundle, Hector Hernandez, John Hamberger, John Catalan, Changhong Zhou, Svetlana Stegalkina, Andrea Tiffany, Harry Kleanthous, Simon Delagrave, Stephen F. Anderson

**Affiliations:** Discovery North America, Sanofi Pasteur, Cambridge, Massachusetts, United States of America; Cincinnati Children's Hospital Medical Center, United States of America

## Abstract

Genital herpes is a sexually transmitted infection (STI) caused by herpes simplex virus 2 (HSV-2) and to a lesser extent herpes simplex virus 1 (HSV-1). Infection by HSV-2 is life-long and is associated with significant cost to healthcare systems and social stigma despite the highly prevalent nature of the disease. For instance, the proportion of HSV-2 seropositive to seronegative adults is approximately 1 in 5 in the US and greater than 4 in 5 in some areas of sub-Saharan Africa. The replication-defective vaccine strain virus *dl*5-29 was re-derived using cells appropriate for GMP manufacturing and renamed ACAM529. Immunization with *dl*5-29 was previously reported to be protective both in mice and in guinea pigs, however these studies were performed with vaccine that was purified using methods that cannot be scaled for manufacturing of clinical material. Here we describe methods which serve as a major step towards preparation of ACAM529 which may be suitable for testing in humans. ACAM529 can be harvested from infected cell culture of the *trans*-complementing cell line AV529 clone 19 (AV529-19) without mechanical cell disruption. ACAM529 may then be purified with respect to host cell DNA and proteins by a novel purification scheme, which includes a combination of endonuclease treatment, depth filtration, anion-exchange chromatography and ultrafiltration/diafiltration (UF/DF). The resultant virus retains infectivity and is ∼ 200-fold more pure with respect to host cell DNA and proteins than is ACAM529 purified by ultracentrifugation. Additionally, we describe a side-by-side comparison of chromatography-purified ACAM529 with sucrose cushion-purified ACAM529, which shows that both preparations are equally immunogenic and protective when tested *in vivo*.

## Introduction

HSV-2 is the primary cause of infectious ulcerative genital disease worldwide [1], [2], [3], [4]. Initially, HSV-2 infects epithelial cells at the mucosal surface. It then establishes latency by retrograde transport along nerve axons to the dorsal root ganglia [5]. During reactivation, the virus travels by anterograde transport to the skin surface where it can cause outward disease symptoms (genital ulcers, nonspecific symptoms or lesions and/or prodrome such as tingling, itching and pain) or may be asymptomatic. Worldwide there are an estimated 23 million new HSV-2 infections per annum [6], which underscores the clinical importance of this viral pathogen. As further evidence to the burden of this disease to US healthcare, Fisman *et al.* estimate that the cost to treat HSV-2 infection could top $2.5 billion per year by 2015 [7] with care centered around oral antiviral treatment and in some cases prevention of transmission with valacyclovir.

Based upon the clear medical need for an effective HSV-2 vaccine, a number of approaches have been tested in the clinic (reviewed by [8]). Examples of randomized, placebo-controlled vaccine trials in humans include inactivated [9], [10], live attenuated [11], [12], [13], subunit [14], [15], [16], [17], [18], DNA [19] and peptide [20] approaches, which have been tested either in the prophylactic or therapeutic setting. The degree of success has been varied, although at least in one case certain populations (HSV-1 and HSV-2 seronegative women) do appear to be protected [14], [16], [21]. Since it is thought that there may be near-universal susceptibility to HSV-2 infection [22] it is essential that there be some means for prevention of disease in sexually active populations. To address the lack of an effective vaccine, a replication defective HSV-2 vaccine strain virus (*dl*5-29, which has since been re-derived and renamed ACAM529 [23]) has been constructed by deleting the U_L_5 and U_L_29 genes from the wild type virus [24]. The vaccine strain virus *dl*5-29 induces a protective immune response *in vivo* in mice and guinea pigs without either replication or establishment of latency [25], [26], [27]. Additionally, *dl*5-29 was shown to be effective in prevention of latent infection in guinea pigs irrespective of HSV-1 serostatus [27]. However, these studies were carried out with vaccine purified using centrifugation-based methods which are not readily scaled for commercial production. Indeed, a number of groups have defined laboratory-scale procedures for purification of herpes viruses based upon centrifugation [28], [29], gradients [30], [31], [32], [33], filtration [34] and affinity chromatography [35], [36].

An essential step towards testing of any live or replication-defective viral vaccine in the clinic involves development of upstream virus production and downstream virus purification procedures to preserve infectivity while allowing for removal of host cell contaminants. As reviewed by Wolf *et al.* [37], cell culture-derived virus particles are of increasing importance medicinally. A variety of downstream processing methods for generation of cell culture derived virus particles have been described (reviewed by [37]). Here we show that preparation of highly pure, functional ACAM529 by a novel chromatography-based purification procedure results in vaccine virus of equal potency *in vivo* to crude vaccine which was prepared by sucrose-cushion ultracentrifugation. Crucial to the development of a robust purification method is the viral harvest method, which here utilizes chemical treatment of infected cells by the sulfated polymeric anion dextran sulfate (DS) to elute the virus from the surface of the cells.

The inhibitory effect of the polyanionic polysaccharide heparin on herpes simplex virus infectivity was first documented in the literature four decades ago [38]. Some twenty years later the mechanism behind this inhibitory effect was interrogated using the synthetic heparin analog dextran sulfate as a substitute [39], [40], [41]. Contrary to what had been originally thought, inhibition of infection by herpes viruses was indeed specific to herpes simplex viruses HSV-1 and HSV-2 as well as some other enveloped viruses, including cytomegalovirus, vesicular stomatitis virus and human immunodeficiency virus [39]. Additionally, at low, non-inhibitory concentrations the glycosaminoglycans heparin, dextran sulfate and chondroitin sulfate act as artificial receptors or co-receptors to mediate HSV infection and can actually increase infectivity [42], [43], [44].

In 1998, O’Keeffe *et al.* showed that heparin and to a lesser extent dextran sulfate could be used as a means for releasing mature vaccine strain disabled infectious single cycle (DISC) virus from monolayers of infected Vero complementing cells [45]. In a subsequent study, O’Keeffe and colleagues described a purification procedure utilizing immobilized heparin as affinity adsorptive chromatographic resin for recovery and purification of the DISC vaccine virus [46]. These early studies described either the primary processing (dextran sulfate release) of the DISC vaccine candidate, or the affinity absorptive recovery of the virus after release of the virus from cells by either hypertonic treatment or treatment with heparin. Here we describe a comprehensive purification procedure with reasonable yields, which is comprised of eluting the candidate vaccine ACAM529 from infected complementing Vero cell (AV529-19) culture using dextran sulfate and then further processing the dextran sulfate-released virus to sufficiently high purity for testing *in vivo* using a combination of endonuclease treatment, depth filtration, anion exchange chromatography and hollow fiber tangential flow filtration (TFF). The resultant vaccine strain virus is highly purified and functional, which is critical in cases where viral infectivity is required for immunogenicity.

## Materials and Methods

### 2.1. Cells, Master Virus Seed and Cell Culture Media

ACAM529 production was accomplished by infection of a monolayer of complementing Vero cells (cell line AV529-19). Complementing cells were obtained as follows: African Green Monkey (Vero) ATCC cell line CCL-81.2 was stably transfected with plasmids pCId.U_L_5, pcDNA.U_L_29 and pSV2neo, which were provided by Dr. David Knipe (Harvard Medical School). Populations of cells were screened and clone AV529-19 was selected for its ability to best complement *dl*5-29 (as ACAM529 was previously known in the literature). The cell line has been grown and maintained in OptiPro (Life Technologies, Carlsbad, CA) supplemented with 4 mM glutamine (Hyclone, Logan, UT) and 10% FBS (Life Technologies) at 37°C in a 5% CO_2_ atmosphere. Cell culture conditions for the purpose of infection and production of ACAM529 are described below. The pre-master virus seed (preMVS) used to produce the ACAM529 master virus seed (MVS) was prepared in several steps from an original stock of *dl*5-29 as follows: the *dl*5-29 virus was propagated using complementing Vero cells, viral genomic DNA was extracted from the resulting virus and provided by Dr. David Knipe (Harvard Medical School) for transfection into AV529-19 cells, and the resulting virus amplified by a single passage. Viral genomic DNA was extracted from this amplified passage and transfected into AV529-19 cells under GLP conditions. The resultant virus was harvested, amplified by one passage, plaque-purified four times, amplified by passaging, and banked as the pre-MVS. The ACAM529 master virus seed (MVS) was prepared and banked under GMP using the pre-MVS and AV529-19.

### 2.2. ACAM529 Upstream Production

Development was undertaken in order to determine upstream growth and infection conditions. Experiments were first performed at the small scale (12-well tissue culture plate and T125 flask) and eventually scaled up to production in 10-layer NUNC cell factories (NCF’s) with a working volume of 2 L and 6,320 cm^2^ cell culture area. For clarity, upstream growth conditions are presented at the NCF scale. A single NCF was seeded with 3.8×10^8^ serum-free AV529-19 cells in OptiPro media supplemented with 4 mM GlutaMAX (Life Technologies) and 500 µg/mL G418 (Life Technologies). The cells were grown at 37°C in a 5% CO_2_ humidified incubator, with a single medium change at 48 h to 1.3 L 40% OptiPro diluted in Dulbecco’s phosphate buffered saline (DPBS) and supplemented with 0.5× cholesterol lipid concentrate (Life Technologies) and 50 mM sucrose. Cells were grown to confluence by incubation for an additional 48 h as above. At 96 h after seeding, the medium was decanted and replaced with 1.3 L of infection medium (40% OptiPro in DPBS with 0.5× cholesterol, 50 mM sucrose) and vaccine inoculum at a multiplicity of infection (MOI) of 0.01. Infection was allowed to proceed at 34°C for 72 h (+/−4 h). Both MOI and time of harvest were optimized to ensure maximal production of ACAM529.

### 2.3. ACAM529 Sucrose Cushion-based Purification Scheme - Microfluidization, Benzonase®, Flat-sheet Tangential-flow Filtration and Ultracentrifugation

In the case where mechanical cell disruption was used to liberate ACAM529 from the biomass, infected cells were detached from the substrate by manual disruption of the monolayer at 72 hours post infection (hpi). Cells were poured from the NCF and a cell pellet was prepared by centrifugation at 1,000×*g*. It was determined that at this point in the procedure it is possible to freeze the ACAM529-containing cell pellet at −80°C without an appreciable loss in titer, for storage prior to processing. The cell pellet from a single NCF was brought to 1 L with stabilization buffer (50 mM potassium glutamate, 10 mM L-histidine, 160 mM NaCl, 10% sucrose, pH 7.0). The cell suspension was processed using a microfluidizer (Microfluidics Corporation, Newton, MA) at 3,000 psi, on ice to mechanically disrupt cells and shear cellular genomic DNA. The solution was adjusted to 5 mM MgCl_2_ and 15,000 units of Benzonase® endonuclease (EMD/Merck, Darmstadt, Germany) were added to the ACAM529-containing solution. The Benzonase® reaction was allowed to proceed at 25°C for 4 h. The cellular lysate was then clarified by centrifugation at 5,000×*g* for 30 min at 4°C. Subsequently, the cleared cellular lysate was concentrated by flat sheet TFF on a Pellicon® XL50 microfiltration system (Millipore, Bedford, MA). Three Pellicon® XL50 cassettes (Biomax, 30 kDa, polyethersulfone, 50 cm^2^) were mounted on a Labscale™ TFF System (Millipore) using the multi-manifold accessory. The volume of the solution was reduced from ∼ 1100 mL to ∼ 50 mL. Throughout filtration, the inlet pressure was maintained at 30 psi, while back pressure was increased from 1 to 8 psi as needed to achieve a practical flux. Finally, the TFF retentate was subjected to ultracentrifugation for 4 h at 50,000×*g* and 4°C over a 25% sucrose cushion, prepared in DPBS with CaCl_2_ and MgCl_2_. The ACAM529-containing pellet was finally resuspended in stabilization buffer with 20% sucrose prior to being aliquoted, flash frozen on dry ice/ethanol and stored at −80°C.

### 2.4. ACAM529 Chromatography-based Purification Scheme - Dextran Sulfate Harvest, Benzonase®, Depth Filtration, Column Chromatography and Hollow Fiber TFF for UF/DF

When cells exhibited ∼ 100% cytopathic effect (CPE), as characterized by rounding of the cells while remaining attached (72 hpi), ACAM529 was harvested by treatment with dextran sulfate. Initial development of the ACAM529 viral harvest (dextran sulfate (DS) elution) procedure was performed in 12-well tissue culture plates. Parameters which were tested and/or optimized include: buffer (conditioned culture media, citrate and glutamate+histidine), pH (6.5–7.5), DS concentration (0–500 µg/mL), dextran sulfate molecular weight (5–5,000 kDa), degree of sulfation (dextran sulfate *vs.* heparin), temperature (34 and 37°C), osmolality (0–30% sucrose), agitation (+/−), time (3, 5, 8 and 24 h) and timing (2–3 days post infection (dpi)) of release.

Results from a single representative screening experiment are presented in this report: For screening purposes, infection medium was decanted from AV529-19 cells in 12-well plates at either day 2 or day 3 after infection. The medium was replaced with 600 µL of dextran sulfate elution buffer: stabilization buffer at pH 7.5 containing 0, 25, 50, 100, 200 or 500 µg/mL dextran sulfate (MW ∼ 5 kDa) (Polydex Pharmaceuticals, Toronto, Canada). The plates were incubated at 34°C for 3, 5, 8 or 24 h before harvest. Dextran sulfate-released ACAM529 was prepared for potency testing by centrifugation at 1,000×*g* to remove cells and cellular debris.

The optimized procedure for large (NCF) scale release of ACAM529 from AV529-19 cells was as follows: at 3 dpi the infection medium was decanted; a sterile, disposable funnel was placed into the NCF inlet port, and 600 mL of pre-warmed (34°C) dextran sulfate elution buffer (stabilization buffer at pH 7.5 containing 100 µg/mL of DS with a MW of ∼ 5 kDa) was poured into the NCF. The NCF was incubated 24 h in a humidified, 5% CO_2_ incubator at 34°C without agitation. After 24 h of incubation, the elution buffer was decanted from the NCF and clarified by centrifugation for 20 min at 1,000×*g*. It was determined that at this point in the procedure it is possible to freeze ACAM529-containing harvest fluid at −80°C without a loss in titer.

If previously frozen, the bulk harvested material was removed from the −80°C freezer and quick-thawed by placing in a 37°C water bath with frequent agitation. The solution was adjusted to 5 mM MgCl_2_ and ninety units of Benzonase® were added per mL of ACAM529-containing solution. The Benzonase® reaction was allowed to proceed at 25°C, with gentle agitation (80 rpm) for 4–6 h. Prior to performing chromatography, the Benzonase®-treated solution was further clarified by depth filtration (0.65 µm SartoScale SartoPure PP2, Sartorius Stedim, Göettingen, Germany) to remove any remaining cellular debris or aggregated material that might clog the chromatographic membrane. The depth filtration manifold was assembled using ¼” sterile flange fittings (tri-clover to hose-tail barb) with the requisite gaskets and tri-clover clamps as well as size 24 silicone MasterFlex tubing (Cole-Parmer, Court Vernon Hills, IL). The entire manifold was autoclaved for 25 min at 121°C dry cycle, as recommended by the manufacturer. The Benzonase®-treated sample was passed though the autoclaved depth filter at 50 mL/min by peristaltic pump (MasterFlex, Cole Parmer), without pretreatment or preequilibration of the membrane.

At the NCF scale, the chromatography flowpath was assembled with size 25 silicone MasterFlex (Cole Parmer) tubing and 1½” sterile flange fittings (tri-clover to host-tail barb) with associated gaskets and tri-clover clamps. The flowpath, including the chromatographic membrane was prepared and chemically sterilized as per the manufacturers’ instructions. Briefly, the membrane (10 mL Mustang® Q capsule, Pall Corporation, Port Washington, NY) was wet with filter-sterilized reverse osmosis deionized (RODI) water while venting. Subsequently, the membrane was sterilized and preconditioned at 100–200 mL/min with 500 mL 0.5 M NaOH and 500 mL 1 M NaCl, respectively. Chromatography running buffer was comprised of stabilization buffer at pH 7.0 with the concentration of sodium chloride described for each step. The membrane was equilibrated with low salt (0.16 M NaCl) column equilibration buffer, until the pH and conductivity of the outlet stream matched that of the original buffer (∼1.5 L of buffer). All subsequent chromatography steps were performed at 60 mL/min. Initially, the ACAM529-containing sample was loaded onto the membrane, and a flowthrough fraction was collected, the membrane was then washed with equilibration buffer until the UV (280 nm) trace returned to baseline and a two-step salt elution was performed. Pre-elution of impurities was performed with 0.7 M NaCl-containing buffer. The pure, infectious, ACAM529-containing fraction was eluted from the membrane with 2 M NaCl-containing buffer. Originally, infectious virus was eluted from the membrane in two steps (1.4 and 2 M NaCl). Subsequent analysis revealed that the two steps had comparable purity, and the higher salt concentration (2 M NaCl) was chosen to elute ACAM529 in a single higher titer step. All fractions were collected manually while observing the absorbance at 280 nm on a chart recorder. An in-line digital pressure monitor was used to ensure that the pressure remained below 94 psig (maximum operating pressure).

Finally, the 2 M NaCl elution fraction was concentrated (5-10-fold by volume) and buffer-exchanged into the final formulation by diafiltration against 3–5× the volume of stabilization buffer containing 20% sucrose. This was performed by hollow fiber tangential flow filtration (100 kDa MWCO, 85 cm^2^, polysulfone hollow fiber TFF module, Spectrum Laboratories, Rancho Dominguez, CA) on a Kros-Flo® Research II system, although in initial experiments (labeled preparations A-D for the purposes of this report) flat sheet TFF was performed as described in the previous section. In order to minimize shear, the lowest suggested flow rate was utilized (130 mL/min, which equates to a shear rate of 4,000 s^−1^). The transmembrane pressure (TMP) was kept below 4 psi throughout the diafiltration process to minimize formation of a gel layer, which could impede fluid flux. As before, the final ACAM529-containing material was aliquoted, flash frozen on dry ice/ethanol and stored at −80°C. Due to the large size of the HSV-2 virus particle (180–200 nm), sterile filtration of the final material is not possible. For this reason, all manipulations must be performed under aseptic conditions.

The chromatogram presented in this report corresponds with a small-scale experiment performed on representative material with a Mustang® Q coin (0.35 mL) on an ÄKTA Explorer (GE Healthcare, Piscataway, NJ). The chromatographic parameters were the same as described above, except that the flow rate was 3 mL/min and step elution was performed automatically over 30 column volumes (CV). Additional modifications to small scale studies presented here include the use of dead end filtration (0.8 µm, 25 mm, supor membrane, syringe filter (Pall Corporation)) as a substitute for depth filtration and dialysis in slide-a-lyzer® cassettes (Thermo-Fisher Scientific (Pierce Protein Research Products), Rockford, IL) MWCO 10–20 kDa for buffer exchange instead of TFF.

While a wide variety of alternative approaches were attempted, ultimately dextran sulfate release, Mustang® Q and hollow fiber TFF were used for purification. Examples of chromatography chemistries and resins which were considered inadequate for reasons of yield or purity after assessment at small (20–50 mL) scale are as follows: HiTrap™ Heparin HP (GE Healthcare), Cellufine® Sulfate (CHISSO Corporation, Tokyo, Japan), HiTrap™ Capto™ Q (GE Healthcare), GigaCap® Q (TOSOH, Yamaguchi, Japan), UNOsphere™ Q (Bio-Rad, Hercules, CA), Fractogel® [DEAE, TMAE and TMAE HiCap] (EMD/Merck), CIM® [Q, DEAE, EDA, and SO_3_] (BIASeparations, Villach, Austria), etc.

### 2.5. Titration of ACAM529

Infectivity of ACAM529 was assessed by titration of samples on the complementing cell line. 12-well tissue culture plates were seeded one day prior to inoculation with 4×10^5^ cells per well. Samples were serially diluted, plated and incubated 1 h, 37°C, 5% CO_2_, with gentle rocking every 15 min. One mL of methyl cellulose overlay medium (in DMEM supplemented with L-glutamine, heat-inactivated FBS and antibiotics) was added to each well and the plates were incubated 48 h. Plaques were visualized by staining with 1% crystal violet in 70% methanol. After manual counting of plaques, titers were represented as plaque forming units (PFU)/mL.

### 2.6. ACAM529 Purity Assays (ELISA, qPCR and PicoGreen dsDNA Assay)

Commercially available ELISA was utilized to determine the purity of process retains as well as of purified ACAM529. ELISAs against Benzonase® (EMD/Merck), Vero Host Cell Protein (HCP) (Cygnus Technologies, Southport, NC) and dextran sulfate (Lifespan Technologies, Salt Lake City, UT) were used. Assays were performed as per the manufacturer’s instructions, except that the following diluents were used in the sample preparation in cases where the diluent was not specified: Vero HCP ELISA (50 mM Tris, 0.1 M NaCl, 8 mg/mL bovine serum albumin, pH 7.0) and DS ELISA (1×phosphate buffered saline (PBS), pH 7.4). Assay specific limits of detection (LOD) are 0.1 ng/mL (Benzonase®), 2 ng/mL (Vero HCP) and 0.003 µg/mL (DS).

Residual Vero DNA testing of ACAM529 samples was contracted to WuXI AppTec, Inc. (Philadelphia, PA) on a sample-by-sample basis. Briefly, the assay is a quantitative PCR (qPCR)-based GLP/GMP assay using ABI Fast 7500 Taqman® technology. Results were provided in the form of a final report, indicating the amount of residual Vero DNA for three nested ribosomal RNA amplicons of 102, 401 and 765 base pairs (bp). For the purposes of this study, the assay was performed at the research level (non-GMP). Data representing the 102 bp amplicon are presented in the results section of this report. The limit of quantitation (LOQ) for this assay is ≤1 pg/µL. Some samples were assayed for dsDNA content using the Quant-iT™ PicoGreen dsDNA Assay Kit (Invitrogen) as per the manufacturer’s instructions.

### 2.7. Ethics Statement

This study was carried out in strict accordance with the recommendations in the Guide for the Care and Use of Laboratory Animals of the National Institutes of Health. The protocol was approved by the Sanofi Pasteur Institutional Animal Care and Use Committee (Protocol Number: 2009-02-01).

### 2.8. Animal Procedures and Analysis

Subcutaneous (sc) immunization of female BALB/c mice (Charles River, Wilmington, MA) 6–7 weeks old was performed in the scruff of the neck on days 0 and 21 of the study. On day 0, animals were injected with 100 µL sterile PBS (group 3) or with 1×10^6^ PFU of ACAM529, either sucrose cushion-purified (group 1) or chromatography-purified (group 2), diluted to 100 µL with sterile PBS. On day 34 of the study, mice were injected sc with 2 mg depot medroxyprogesterone acetate (Depo-Provera, DMPA) (SICOR Pharmaceuticals Inc., Irvine, CA) in PBS. Seven days later, mice were challenged intravaginally with 50 LD_50_ (8×10^4^ PFU) of HSV-2 strain 333 in 20 µL with a positive displacement pipet. HSV-2 strain 333 was a generous gift from Dr. Jeffrey Cohen (NIAID, Medical Virology Section). Animals were observed for 14 days post challenge. Mice were euthanized upon observation of purulent genital lesions. Animals were bled on days 18, 35 and 41 of the study.

Endpoint ELISA titers against HSV-2 purified viral lysate (Advanced Biotechnologies, Colombia, MD) were determined for serum from day 35 samples. Plates (96 well Maxisorp, Nalge NUNC International, Rochester, NY) were coated with 100 µL/well of HSV-2 viral lysate at a concentration of 2 µg/mL. Serum IgG was detected with 1∶2,000 biotin-anti-mouse IgG Fc (Sigma-Aldrich, Saint Louis, MO) diluted in 1% BSA, 0.05% Tween in PBS. Time resolved fluorescence (TRF) signal was measured using a Victor II fluorometer (Perkin Elmer, Waltham, MA) after addition of 0.1 µg/mL Delfia europium-streptavidin conjugate in Delfia Assay Buffer.

## Results

### 3.1. Drawbacks of the Ultracentrifugation Method

Preparation of the vaccine strain virus ACAM529 by laboratory-scale virological methods (including sucrose cushion ultracentrifugation) results in crude material with greater than 2 µg of residual host cell DNA per 1×10^7^ PFU of ACAM529 (the World Health Organization limit is 10 ng DNA per human dose). In contrast, using the methods described here we are able to prepare ACAM529 such that the amount of residual Vero DNA is below 10 ng per 1×10^7^ PFU of ACAM529. Additionally, ACAM529 prepared by the methods described here is 200-fold more pure with respect to host cell proteins and 2 orders of magnitude more pure with respect to the process stream contaminant dextran sulfate. Of paramount importance is that highly pure ACAM529 is functionally indistinguishable from crude preparations by plaque assay titration as well as *in vivo*.

### 3.2. ACAM529 can be Chemically Eluted from the Surface of Infected Trans-complementing Cells with Dextran Sulfate

**Figure 1:**
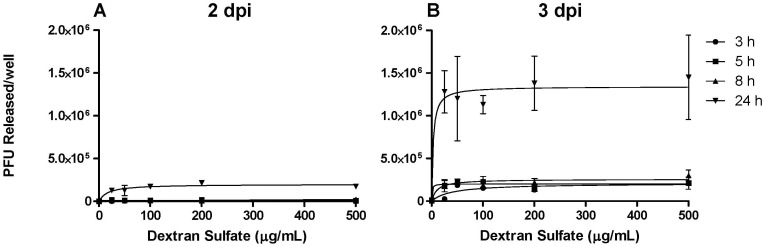
Chemical elution of ACAM529 from the surface of infected AV529-19 complementing cells. Medium was decanted from confluent, infected AV529-19 cells grown in 12-well tissue culture plates and replaced with stability buffer containing 10% sucrose and 25–500 µg/mL dextran sulfate. At time points, 3 h (•), 5 h (▪), 8 h (▴) and 24 h (▾), after the start of the dextran sulfate elution the samples were processed by centrifugation at 1,000×*g* and freezing at −80°C prior to titration by plaque assay. The results presented on the ordinate represent the titer (PFU/mL) of the DS release supernatant, error bars represent the standard deviation of the mean.

When mechanical cell disruption was used prior to chromatographic separation the resultant virus was high in dsDNA (0.33 or 2.0 µg/mL by dsDNA assay, for samples which either were or were not treated with Benzonase®, respectively) and the recovery of the virus was poor; 12% recovery for Benzonase®-treated samples and 39% for those which had not been treated with Benzonase® (Table 1). This highlighted the necessity for using non-mechanical means to harvest the virus from the production cells. Figure 1 shows the result of a single representative screening experiment to address whether ACAM529 could be eluted from the surface of AV529-19 cells using DS. Cells were grown in 12-well tissue culture plates and stability buffer containing sucrose was used to dissolve the dextran sulfate. When cells at 3 dpi were incubated with DS, one could detect infectious material in the supernatant after 24 h of incubation when ≥25 µg/mL of DS was used (Figure 1B). A concentration of 100 µg/mL DS was selected for further studies.

**Table 1:** Virus step yield (PFU) from small-scale screening of anion exchange purification conditions (harvest method, chromatography resins, etc.).

Harvest	Resin	Benzonase®	Step Yield
Microfluidization	CIM® DEAE	−	5%
Microfluidization	CIM® Q	−	2%
Sonication	Capto™ Q	−	31%
Sonication	Capto™ Q	+	12%
Sonication	HiTrap™ DEAE FF	−	10%
Sonication	Mustang® Q	−	39%
Sonication	Mustang® Q	+	12%
Dextran Sulfate	Mustang® Q	−	95%
Dextran Sulfate	Mustang® Q	+	75%
Dextran Sulfate	Fractogel® DEAE	+	61%
Dextran Sulfate	Fractogel® TMAE	+	59%
Dextran Sulfate	Fractogel® TMAE HiCap	+	67%
Dextran Sulfate	CIM® DEAE	+	15%
Dextran Sulfate	CIM® Q	+	6%
Dextran Sulfate	CIM® EDA	+	6%
Dextran Sulfate	UNOsphere™ Q	+	22%
Dextran Sulfate	Capto™ Q	+	65%
Dextran Sulfate	GigaCap® Q	+	44%

### 3.3. DNA Reduction

Benzonase® endonuclease treatment of ACAM529 substantially increases the purity of infectious virus after chromatography by Mustang® Q, as observed by agarose gel electrophoresis of purified virus with or without Benzonase® treatment (data not shown). Additionally, at the small (∼ 20 mL) scale, dead end filtration of material which had not been treated by Benzonase® resulted in a ∼ 50% recovery of infectious titer, whereas filtration of material post-Benzonase® treatment resulted in a much better (∼ 100%) step yield. In contrast, more of the non-Benzonase®-treated material was recovered by Mustang® Q chromatography than Benzonase®-treated; ∼ 95% as compared to ∼ 75% (Table 1). Overall yield for each process was similar, but losses were sustained at different points along the downstream purification train. Based upon these observations, clarification of the bulk dextran sulfate-released material by depth or dead-end filtration should be performed *after* endonuclease treatment to prevent fouling of the filter and loss of titer. Additionally, Benzonase® treatment should be performed to ensure that final purified material is low in contaminating DNA.

### 3.4. Mustang® Q Anion Exchange and Hollow Fiber TFF for Purification of ACAM529

**Figure 2:**
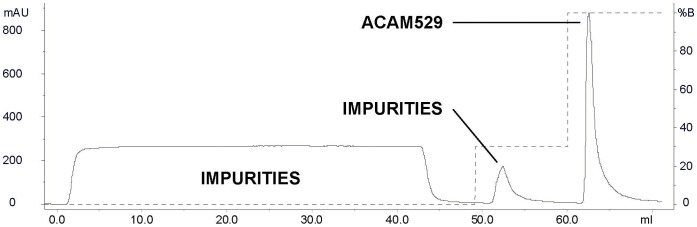
Chromatographic profile during small scale (0.35 mL Mustang® Q coin) bind-and-elute purification of ACAM529 by membrane-based anion exchange. Solid line represents the elution profile for absorbance at 280 nm, whereas the dotted line represents the concentration of salt as a percentage of the high salt buffer (Buffer B, 2 M NaCl). During the sample loading phase (0–50 mL) ACAM529 binds the solid support, while unbound impurities pass through the column and are collected as the flowthrough fraction. Pre-elution of bound, non-viral protein impurities is achieved by applying a 700 mM NaCl (30% B) step over 30 column volumes (50–60 mL). Bound ACAM529 is eluted from the column by step-wise increase of the salt concentration to 2 M NaCl (100% Buffer B) over 30 CV (60–70 mL).

**Figure 3:**
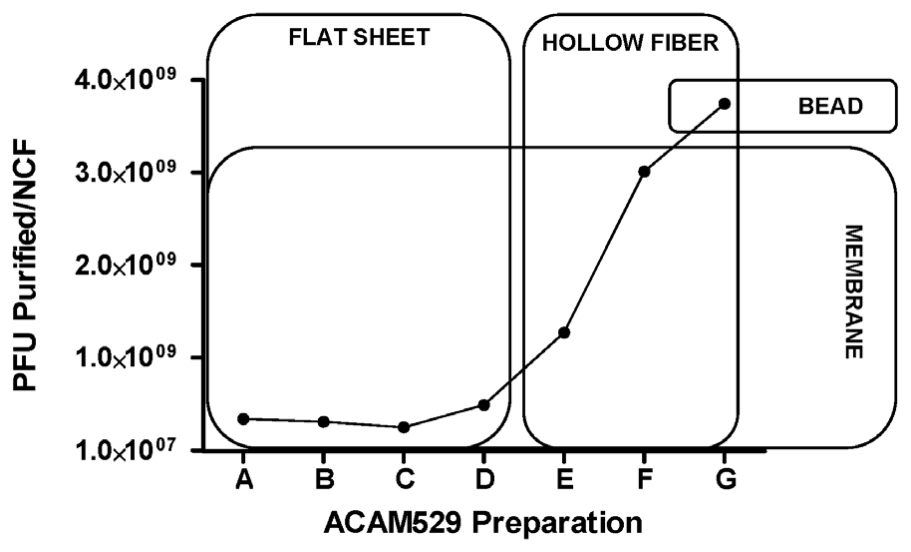
Optimization of purification conditions to achieve a yield of ∼ 4×10^9^ PFU per NUNC cell factory. Each of the points on the curve represents the yield from an entire purification, starting with material which had been dextran sulfate-released from a single NCF of infected cell culture. ACAM529 Purifications A–G were performed sequentially, with optimization of purification steps to improve yield and purity. The overall yield (y-axis; PFU purified per NCF) increased with time as purification conditions were optimized. Flat sheet TFF was originally tested as an option for concentration and formulation of the partially purified vaccine virus (ACAM529 Preparations A–D). Low step yield for flat sheet TFF (∼20–40%) led us to test hollow fiber TFF as an alternative (Preparations E–G), with dramatic improvement (∼70–100% step yield) of recovery of infectious virus. Additionally, the high-capacity strong anion exchanger, Fractogel TMAE HiCap (BEAD, Preparation G) was tested as an alternative to Mustang® Q (MEMBRANE, Preparations A–F) as the bind-and-elute chromatography step.

**Figure 4:**
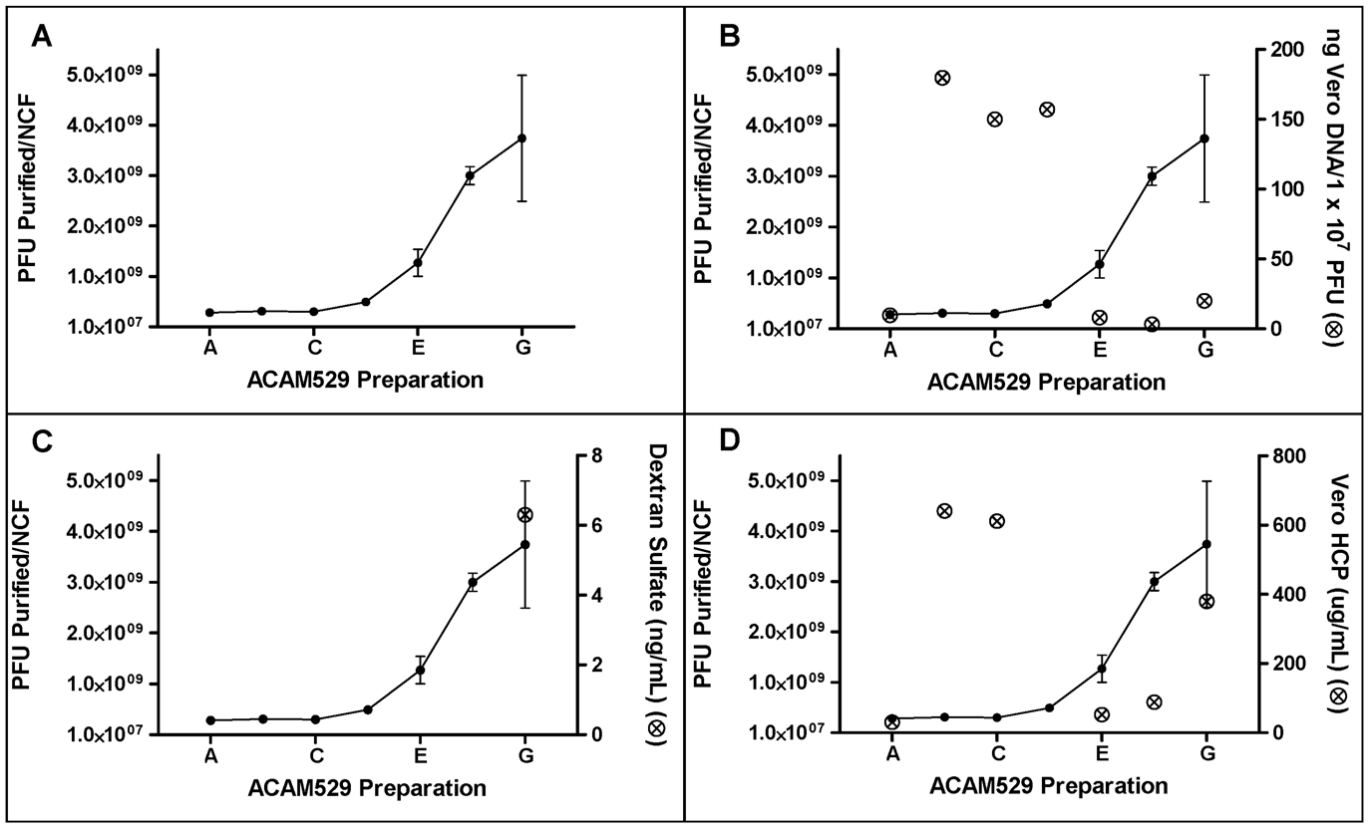
Purity of ACAM529 virus preparations. Superimposition of yield (panel A) with purity (⊗) (panels B–D) results for ACAM529 Preparations A–G. The right y-axes in panels B–D represent results from purity assays: residual Vero DNA qPCR, LOQ ≤1 pg/µL (B), DS ELISA, LOD 3 ng/mL (C) and Vero HCP ELISA, LOD 2 ng/mL (D). In panel C, for preparations A–F, and panel D, preparation D, where no purity data point is present, the amount of impurity in the final material was below the assay-specific LOD. In all cases, the purity of Preparation F exceeded that of Preparation G, exemplifying why the Preparation F conditions were decided upon for use as the final purification scheme.

Determination of the appropriate chromatographic support for bind-and-elute chromatography was performed in a series of small-scale screening experiments, with the primary intention of attaining maximum yield of infectious virus in the eluted fraction. Table 1 shows a non-exhaustive list of yields from such screening experiments. Since the maximum yield was obtained with the Mustang® Q membrane, this was chosen for scale up to purification of material from a single NCF. Figure 2 shows the chromatographic profile for elution of ACAM529 (DS-harvested, Benzonase®-treated, and dead-end filtered) prior to loading onto the Mustang® Q coin (0.35 mL membrane format for screening studies). The ACAM529 containing fraction is eluted from the support at 100% B or 2 M NaCl, as labeled. Overall yield (presented as the number of PFU purified per NCF) for ACAM529 purifications from optimization experiments (labeled Preparations A-G) are presented as Figure 3. The overall yield increased as chromatography and other purification conditions were optimized. Major changes which positively impacted the yield are highlighted by boxes. Yield was effectively doubled by switching from flat sheet TFF with the Pellicon XL system to hollow fiber TFF with the Kros-Flo system (Figure 3 horizontal boxes). It is thought that the increase in yield is due to a lower shear force being generated by open-channel flow as opposed to the flat sheet system where a turbulence generating screen in the flow path acts to maximize flux by minimizing formation of a gel layer. The hollow fiber TFF module which was used in the experiments presented here had a MWCO of 100 kDa, we also tested a 500 kDa MWCO cassette but yield was consistently lower than what is described here (data not shown). Replacement of the Mustang® Q membrane-based anion exchanger with a bead-based tentacle resin (Fractogel TMAE HiCap, EMD Merck) resulted in a non-significant increase in yield and lower purity (compare ACAM529 Preparations F and G in Figure 4B–D).

### 3.5. Purity and Yield of ACAM529 Purified by a Chromatography-based Purification Scheme

Mustang® Q-purified material (ACAM529 Preparation F) contained less Vero residual DNA (Figure 4B), dextran sulfate (Figure 4C) and Vero HCP (Figure 4D) than the Fractogel TMAE HiCap purified material (ACAM529 Preparation G). To look more specifically at the benefits to using each step in the purification train, Tables 2 and 3 highlight the yield and purity results for each step of the ACAM529 Preparation A purification. As previously mentioned, improvements to the yield (Table 2 column 2) were made by switching from flat sheet to hollow fiber TFF. Depth filtration appears to partially remove dextran sulfate from the feed stream, the rest of which is removed during chromatography (Table 2, column 6). Also, as was expected, Benzonase® was removed during chromatography as it does not bind to anion exchangers at neutral pH (Table 2, column 5). Although we were unable to determine the amount of Vero DNA in the starting material due to 100% interference of the qPCR signal by the sample (as measured by an internal *E. coli* DNA spike control), we were able to show that after Benzonase® treatment, depth filtration and chromatography, the amount of Vero DNA in the sample was less than the WHO limit per proposed human dose of vaccine (Table 2 column 3). Finally, the majority of Vero HCP was removed during chromatography (flowthrough, wash and pre-elution; Table 2 column 4). Inspection of the purification factor (PFU per mg of Vero HCP) for ACAM529-containing fractions shows a 250-fold purification of ACAM529 with respect to Vero HCP (Table 3).

**Table 2:** Purity and step yield of ACAM529 (Preparation A) process retains.

Retain	Yield	DNA	Vero HCP	Benzonase®	DS
	(%)	(ng/1×10^7^ PFU)	(µg/mL)	(ng/mL)	(µg/mL)
Start	45	a	75	<LOD	28
Benzonase®	85	a	127	52	27
Depth Filter	108	nd	104	51	11.25
Mustang® Q FT	0	nd	91	47	<LOD
Mustang® Q Wash	0	nd	7	4	<LOD
Mustang® Q Step 1	0.1	nd	9	<LOD	<LOD
Mustang® Q Step 2	31	<10	6	<LOD	<LOD
Mustang® Q Step 3	29	<10	3	<LOD	<LOD
TFF Permeate	0	nd	0	<LOD	<LOD
TFF Retentate	38	9.74	30	<LOD	<LOD

a We were unable to determine the amount of Vero DNA in the starting material, as even at high dilutions there was 100% interference of qPCR signal by the sample (dextran sulfate and/or Benzonase®).

**Table 3:** The chromatography-based ACAM529 purification process results in 250-fold purification factor with respect to Vero HCP.

Retain	Vero HCP			Purification Factor
	(µg/mL)	(mg)	(PFU/mg)	
Start	75	75	1.5×10^5^	1x
Benzonase®	127	75	1.2×10^5^	1x
Depth Filter	104	60	1.7×10^5^	1x
Mustang® Q FT	91	53	0	–
Mustang® Q Wash	7	3	0	–
Mustang® Q Step 1	9	1	3.4×10^4^	–
Mustang® Q Step 2	6	1	9.2×10^6^	60x
Mustang® Q Step 3	3	0.3	3.1×10^7^	200x
TFF Permeate	<LOD	–	–	–
TFF Retentate	30	0.5	3.7×10^7^	250x

### 3.6. Chromatography-purified ACAM529 is as Immunogenic and Protective as Sucrose Cushion-prepared ACAM529 in vivo

**Figure 5:**
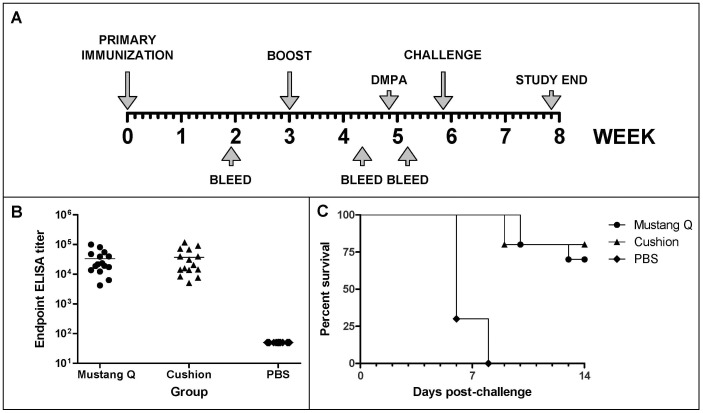
Chromatography-purified ACAM529 is as immunogenic and protective as cushion-purified ACAM529. Panel A is a schematic representation of the animal study schedule, long labeled arrows represent viral inoculations (immunizations were performed sc and challenge was intravaginal) short arrows symbolize bleeds, hormone injection (DMPA = depot medroxyprogesterone acetate or Depo-Provera) and the study end day, as indicated. Panel B shows endpoint ELISA titers against a commercially available, purified HSV-2 viral lysate for immunized mice and Panel C depicts survival of animals as a % of the total (n = 15 animals). Mice were immunized either with Mustang® Q (•)- or sucrose cushion (▴)-purified ACAM529 or a placebo (♦)(PBS). Both vaccine preparations elicited similar anti-HSV-2 ELISA titers (Kruskal-Wallis Test P = 0.99) and similar levels of protection against severe virus challenge with wild type HSV-2 strain 333 **(**Mantel-Cox Test P<0.0001).

Female BALB-c mice were immunized subcutaneously with two doses of ACAM529 prepared either by chromatography (Preparation F) or by sucrose cushion. A lethal challenge study was carried out as schematized in Figure 5A. Serum from blood taken one week after the second and final vaccine dose was tested for IgG response against a commercially available viral lysate. After two immunizations, both preparations elicit a similar anti-HSV-2 IgG response (Figure 5B; P = 0.99, one way ANOVA, Kruskal-Wallis test). Two weeks after the last immunization, animals were treated with medroxyprogesterone and, seven days later, given a 50×LD50 intravaginal challenge with wild type HSV-2 strain 333 (Figure 5C). The cushion-prepared and chromatography-purified vaccine afforded statistically equivalent protection of 80% and 70%, respectively, while mock immunization resulted in complete lethality (0% survival; P<0.0001). A subsequent study with chromatography-purified ACAM529 revealed that complete protection from challenge was achieved when immunization was performed intramuscularly [23].

## Discussion

Vaccination against HSV-2 remains a challenge despite the many, predominantly subunit-based candidates which have been tested in the clinic [8], [9], [10], [11], [12], [14], [15], [16], [17], [18], [19], [20], [21]. ACAM529 is a replication-defective prophylactic vaccine candidate, which has proven efficacious in mice and guinea pigs [23] [24] [25] [26] [27]. Each of the U_L_5 and U_L_29 genes is necessary for replication of the viral genome [24]. Therefore, recombination of both genes from the complementing cell line into the vaccine genome would be necessary for the vaccine to regain its replication competence. To minimize the chance of homologous recombination between the complementing genes and the vaccine genome during manufacturing, all flanking sequences of the HSV-1 U_L_5 and U_L_29 genes in the production cell line were removed except for a 109 bp segment in the 3′ untranslated region of U_L_5 having 72% identity with the vaccine genome.

In an initial evaluation of its phenotypic stability, the vaccine was passaged 17 times in the complementing cell line under conditions selecting for rapid replication. The hypothesis was that presence of even trace quantities of replication-competent virus arising from a hypothetical recombination event would be amplified by passaging. Plaque assays of late passage virus on non-complementing Vero cells failed to reveal the presence of replication-competent virus (data not shown). Deep sequencing of passaged vaccine did not reveal evidence of recombination with either of the complementing genes. Also, as part of the GMP manufacturing process an assay has been developed to identify replication-competent contaminating herpesvirus. To date, such a contaminant has never been observed despite a sensitivity of better than 10^−8^ (*i.e.*, one PFU of wildtype virus can be detected when added to 10^8^ PFU of ACAM529).

In order to advance development of this vaccine beyond animal models and into clinical studies, a scalable process capable of producing material which may be suitable for human use was developed. For the ACAM529 vaccine candidate to proceed to the clinic, additional testing will be necessary. We have shown here that a highly-purified, functional version of ACAM529 can be made by processing of infected complementing Vero cells (AV529-19) by a combination of dextran sulfate elution followed by Benzonase® treatment, depth filtration, anion exchange chromatography and UF/DF. The overall yield for the optimized process is 10–20% of the infectious titer in the starting material, which equates to 1–3×10^9^ PFU per NCF (variability in the vaccine titer in the starting material accounts for the discrepancy between yield (% total) and PFU purified per NCF).

We have observed that the contribution of hydrodynamic shear stress to the loss of infectious virus titer was critical. In nearly all cases, when high-shear systems (closed channel flat sheet TFF and bead-based chromatographic support) are replaced by low-shear unit operations (open channel hollow fiber TFF and membrane-based chromatographic support) more infectious virus is recovered per step of the purification process. Convective liquid flow, as in the case of membrane (Mustang® Q) and monolithic (CIM) chromatographic supports, acts to minimize shear by eliminating flow vortices and turbulent eddies, which occur in the void space in traditional packed bead columns. Shear does not entirely explain recovery as is clear from the difference in yield of infectious virus from the membrane *vs.* the monoliths tested here.

Apart from optimization of chromatography, the most significant process change was from flat-sheet, closed-channel TFF to hollow fiber TFF. This resulted in up to a 10-fold increase in yield without compromising purity. Plaque assay results show that optimization of purification steps results in additional increases in yield also without compromised purity in the case of the Mustang® Q anion exchanger. In contrast, Fractogel TMAE HiCap (a bead-based, strong anion exchanger)-purified material appears less attractive in that the final material contains ∼2-fold more residual DNA and at least 2 orders of magnitude more dextran sulfate. Even still, Fractogel TMAE HiCap might be considered as a candidate chromatography resin since elution conditions might be optimized to improve purity.

The preserved immunogenicity and protective efficacy of the vaccine against a severe lethal challenge in mice supports the validity of this process for production of ACAM529. These data support the use of chromatography-based purification processes for preparation of ACAM529 for *in vivo* testing.

## References

[1] Mertz KJ, Trees D, Levine WC, Lewis JS, Litchfield B, et al (1998) Etiology of genital ulcers and prevalence of human immunodeficiency virus coinfection in 10 US cities. The Genital Ulcer Disease Surveillance Group. J Infect Dis 178: 1795–1798. 9815237 10.1086/314502

[2] Mertz KJ, Weiss JB, Webb RM, Levine WC, Lewis JS, et al (1998) An investigation of genital ulcers in Jackson, Mississippi, with use of a multiplex polymerase chain reaction assay: high prevalence of chancroid and human immunodeficiency virus infection. J Infect Dis 178: 1060–1066. 9806035 10.1086/515664

[3] Paz-Bailey G, Rahman M, Chen C, Ballard R, Moffat HJ, et al (2005) Changes in the etiology of sexually transmitted diseases in Botswana between 1993 and 2002: implications for the clinical management of genital ulcer disease. Clin Infect Dis 41: 1304–1312. 16206106 10.1086/496979

[4] Pena KC, Adelson ME, Mordechai E, Blaho JA (2010) Genital herpes simplex virus type 1 in women: detection in cervicovaginal specimens from gynecological practices in the United States. J Clin Microbiol 48: 150–153. 19923487 10.1128/JCM.01336-09 PMC2812291

[5] Cunningham AL, Diefenbach RJ, Miranda-Saksena M, Bosnjak L, Kim M, et al (2006) The cycle of human herpes simplex virus infection: virus transport and immune control. J Infect Dis 194 Suppl 1S11–18. 16921466 10.1086/505359

[6] Looker KJ, Garnett GP, Schmid GP (2008) An estimate of the global prevalence and incidence of herpes simplex virus type 2 infection. Bull World Health Organ 86: 805–812, A. 10.2471/BLT.07.046128 PMC2649511 18949218

[7] Fisman DN, Lipsitch M, Hook EW 3rd, Goldie SJ (2002) Projection of the future dimensions and costs of the genital herpes simplex type 2 epidemic in the United States. Sex Transm Dis 29: 608–622. 12370529 10.1097/00007435-200210000-00008

[8] Johnston C, Koelle DM, Wald A (2011) HSV-2: in pursuit of a vaccine. J Clin Invest 121: 4600–4609. 22133885 10.1172/JCI57148 PMC3223069

[9] Kutinova L, Benda R, Kalos Z, Dbaly V, Votruba T, et al (1988) Placebo-controlled study with subunit herpes simplex virus vaccine in subjects suffering from frequent herpetic recurrences. Vaccine 6: 223–228. 2844031 10.1016/0264-410x(88)90215-0

[10] Skinner GR, Turyk ME, Benson CA, Wilbanks GD, Heseltine P, et al (1997) The efficacy and safety of Skinner herpes simplex vaccine towards modulation of herpes genitalis; report of a prospective double-blind placebo-controlled trial. Med Microbiol Immunol 186: 31–36. 9255764 10.1007/s004300050043

[11] Casanova G, Cancela R, Alonzo L, Benuto R, Magana Mdel C, et al (2002) A double-blind study of the efficacy and safety of the ICP10deltaPK vaccine against recurrent genital HSV-2 infections. Cutis 70: 235–239. 12403316

[12] de Bruyn G, Vargas-Cortez M, Warren T, Tyring SK, Fife KH, et al (2006) A randomized controlled trial of a replication defective (gH deletion) herpes simplex virus vaccine for the treatment of recurrent genital herpes among immunocompetent subjects. Vaccine 24: 914–920. 16213066 10.1016/j.vaccine.2005.08.088

[13] Awasthi S, Zumbrun EE, Si H, Wang F, Shaw CE, et al.. (2012) Live Attenuated Herpes Simplex Virus Type 2 Glycoprotein E Deletion Mutant as a Vaccine Candidate Defective in Neuronal Spread. J Virol. 10.1128/JVI.07203-11 PMC3318599 22318147

[14] Bernstein DI, Aoki FY, Tyring SK, Stanberry LR, St-Pierre C, et al (2005) Safety and immunogenicity of glycoprotein D-adjuvant genital herpes vaccine. Clin Infect Dis 40: 1271–1281. 15825029 10.1086/429240

[15] Langenberg AG, Burke RL, Adair SF, Sekulovich R, Tigges M, et al (1995) A recombinant glycoprotein vaccine for herpes simplex virus type 2: safety and immunogenicity [corrected]. Ann Intern Med 122: 889–898. 7755223 10.7326/0003-4819-122-12-199506150-00001

[16] Stanberry LR, Spruance SL, Cunningham AL, Bernstein DI, Mindel A, et al (2002) Glycoprotein-D-adjuvant vaccine to prevent genital herpes. N Engl J Med 347: 1652–1661. 12444179 10.1056/NEJMoa011915

[17] Straus SE, Corey L, Burke RL, Savarese B, Barnum G, et al (1994) Placebo-controlled trial of vaccination with recombinant glycoprotein D of herpes simplex virus type 2 for immunotherapy of genital herpes. Lancet 343: 1460–1463. 7911177 10.1016/s0140-6736(94)92581-x

[18] Straus SE, Wald A, Kost RG, McKenzie R, Langenberg AG, et al (1997) Immunotherapy of recurrent genital herpes with recombinant herpes simplex virus type 2 glycoproteins D and B: results of a placebo-controlled vaccine trial. J Infect Dis 176: 1129–1134. 9359709 10.1086/514103

[19] Cattamanchi A, Posavad CM, Wald A, Baine Y, Moses J, et al (2008) Phase I study of a herpes simplex virus type 2 (HSV-2) DNA vaccine administered to healthy, HSV-2-seronegative adults by a needle-free injection system. Clin Vaccine Immunol 15: 1638–1643. 18784341 10.1128/CVI.00167-08 PMC2583522

[20] Koelle DM, Magaret A, McClurkan CL, Remington ML, Warren T, et al (2008) Phase I dose-escalation study of a monovalent heat shock protein 70-herpes simplex virus type 2 (HSV-2) peptide-based vaccine designed to prime or boost CD8 T-cell responses in HSV-naive and HSV-2-infected subjects. Clin Vaccine Immunol 15: 773–782. 18353920 10.1128/CVI.00020-08 PMC2394846

[21] Belshe RB, Leone PA, Bernstein DI, Wald A, Levin MJ, et al (2012) Efficacy results of a trial of a herpes simplex vaccine. N Engl J Med 366: 34–43. 22216840 10.1056/NEJMoa1103151 PMC3287348

[22] Watson-Jones D, Weiss HA, Rusizoka M, Baisley K, Mugeye K, et al (2007) Risk factors for herpes simplex virus type 2 and HIV among women at high risk in northwestern Tanzania: preparing for an HSV-2 intervention trial. J Acquir Immune Defic Syndr 46: 631–642. 18043318 10.1097/QAI.0b013e31815b2d9c PMC2643092

[23] Delagrave S, Hernandez H, Zhou C, Hamberger JF, Mundle ST, et al (2012) Immunogenicity and Efficacy of Intramuscular Replication-Defective and Subunit Vaccines against Herpes Simplex Virus Type 2 in the Mouse Genital Model. PLoS One 7: e46714. 23071620 10.1371/journal.pone.0046714 PMC3469653

[24] Da Costa X, Kramer MF, Zhu J, Brockman MA, Knipe DM (2000) Construction, phenotypic analysis, and immunogenicity of a UL5/UL29 double deletion mutant of herpes simplex virus 2. J Virol 74: 7963–7971. 10933704 10.1128/jvi.74.17.7963-7971.2000 PMC112327

[25] Da Costa XJ, Jones CA, Knipe DM (1999) Immunization against genital herpes with a vaccine virus that has defects in productive and latent infection. Proc Natl Acad Sci U S A 96: 6994–6998. 10359827 10.1073/pnas.96.12.6994 PMC22033

[26] Hoshino Y, Dalai SK, Wang K, Pesnicak L, Lau TY, et al (2005) Comparative efficacy and immunogenicity of replication-defective, recombinant glycoprotein, and DNA vaccines for herpes simplex virus 2 infections in mice and guinea pigs. J Virol 79: 410–418. 15596834 10.1128/JVI.79.1.410-418.2005 PMC538700

[27] Hoshino Y, Pesnicak L, Dowdell KC, Burbelo PD, Knipe DM, et al (2009) Protection from herpes simplex virus (HSV)-2 infection with replication-defective HSV-2 or glycoprotein D2 vaccines in HSV-1-seropositive and HSV-1-seronegative guinea pigs. J Infect Dis 200: 1088–1095. 19702506 10.1086/605645 PMC3826825

[28] Arens M, Swierkosz E, Dilworth V (1988) Effects of sonication and centrifugation of clinical specimens on the recovery of herpes simplex virus. Diagn Microbiol Infect Dis 11: 137–143. 2854511 10.1016/0732-8893(88)90015-6

[29] Lotfian P, Levy MS, Coffin RS, Fearn T, Ayazi-Shamlou P (2003) Impact of process conditions on the centrifugal recovery of a disabled herpes simplex virus. Biotechnol Prog 19: 209–215. 12573027 10.1021/bp025599g

[30] Goins WF, Krisky DM, Wechuck JB, Huang S, Glorioso JC (2008) Construction and production of recombinant herpes simplex virus vectors. Methods Mol Biol 433: 97–113. 18679619 10.1007/978-1-59745-237-3_6

[31] Sathananthan B, Rodahl E, Flatmark T, Langeland N, Haarr L (1997) Purification of herpes simplex virus type 1 by density gradient centrifugation and estimation of the sedimentation coefficient of the virion. APMIS 105: 238–246. 9137520 10.1111/j.1699-0463.1997.tb00564.x

[32] Sia KC, Wang GY, Ho IA, Khor HY, Miao L, et al (2007) Optimal purification method for Herpes-based viral vectors that confers minimal cytotoxicity for systemic route of vector administration. J Virol Methods 139: 166–174. 17074404 10.1016/j.jviromet.2006.09.023

[33] Szilagyi JF, Cunningham C (1991) Identification and characterization of a novel non-infectious herpes simplex virus-related particle. J Gen Virol 72 (Pt 3): 661–668. 10.1099/0022-1317-72-3-661 1848601

[34] Knop DR, Harrell H (2007) Bioreactor production of recombinant herpes simplex virus vectors. Biotechnol Prog 23: 715–721. 17461549 10.1021/bp060373p

[35] Jiang C, Ataai M, Ozuer A, Krisky D, Wechuck J, et al (2006) Inactivation of herpes simplex type 1 gene vector on immobilized metal affinity chromatography: oxidative damage by hydroxyl free radicals and its prevention. Biotechnol Bioeng 95: 48–57. 16673413 10.1002/bit.20943

[36] Jiang C, Wechuck JB, Goins WF, Krisky DM, Wolfe D, et al (2004) Immobilized cobalt affinity chromatography provides a novel, efficient method for herpes simplex virus type 1 gene vector purification. J Virol 78: 8994–9006. 15308696 10.1128/JVI.78.17.8994-9006.2004 PMC506967

[37] Wolf MW, Reichl U (2011) Downstream processing of cell culture-derived virus particles. Expert Rev Vaccines 10: 1451–1475. 21988309 10.1586/erv.11.111 PMC7103681

[38] Nahmias AJ, Kibrick S (1964) Inhibitory effect of heparin on herpes simplex virus. J Bacteriol 87: 1060–1066. 4289440 10.1128/jb.87.5.1060-1066.1964 PMC277146

[39] Baba M, Snoeck R, Pauwels R, de Clercq E (1988) Sulfated polysaccharides are potent and selective inhibitors of various enveloped viruses, including herpes simplex virus, cytomegalovirus, vesicular stomatitis virus, and human immunodeficiency virus. Antimicrob Agents Chemother 32: 1742–1745. 2472775 10.1128/aac.32.11.1742 PMC175964

[40] Neyts J, Reymen D, Letourneur D, Jozefonvicz J, Schols D, et al (1995) Differential antiviral activity of derivatized dextrans. Biochem Pharmacol 50: 743–751. 7575633 10.1016/0006-2952(95)00193-4

[41] Piret J, Lamontagne J, Bestman-Smith J, Roy S, Gourde P, et al (2000) In vitro and in vivo evaluations of sodium lauryl sulfate and dextran sulfate as microbicides against herpes simplex and human immunodeficiency viruses. J Clin Microbiol 38: 110–119. 10618073 10.1128/jcm.38.1.110-119.2000 PMC86033

[42] Banfield BW, Leduc Y, Esford L, Visalli RJ, Brandt CR, et al (1995) Evidence for an interaction of herpes simplex virus with chondroitin sulfate proteoglycans during infection. Virology 208: 531–539. 7747425 10.1006/viro.1995.1184

[43] Dyer AP, Banfield BW, Martindale D, Spannier DM, Tufaro F (1997) Dextran sulfate can act as an artificial receptor to mediate a type-specific herpes simplex virus infection via glycoprotein B. J Virol. 71: 191–198. 10.1128/jvi.71.1.191-198.1997 PMC191039 8985338

[44] Yeung SN, Bockhold K, Tufaro F (1999) Efficient infection of mature skeletal muscle with herpes simplex virus vectors by using dextran sulfate as a co-receptor. Gene Ther 6: 1536–1544. 10490762 10.1038/sj.gt.3300980

[45] O’Keeffe R, Johnston MD, Slater NK (1998) The primary production of an infectious recombinant Herpes Simplex Virus vaccine. Biotechnol Bioeng 57: 262–271. 10099202 10.1002/(sici)1097-0290(19980205)57:3<262::aid-bit2>3.0.co;2-f

[46] O’Keeffe RS, Johnston MD, Slater NK (1999) The affinity adsorptive recovery of an infectious herpes simplex virus vaccine. Biotechnol Bioeng 62: 537–545. 10099562 10.1002/(sici)1097-0290(19990305)62:5<537::aid-bit5>3.0.co;2-1

